# Enzymatic Construction of DARPin-Based Targeted Delivery Systems Using Protein Farnesyltransferase and a Capture and Release Strategy

**DOI:** 10.3390/ijms231911537

**Published:** 2022-09-29

**Authors:** Yi Zhang, Yiao Wang, Safak Uslu, Sneha Venkatachalapathy, Mohammad Rashidian, Jonas V. Schaefer, Andreas Plückthun, Mark D. Distefano

**Affiliations:** 1 Department of Chemistry, University of Minnesota, Minneapolis, MN 55455, USA; 2 Department of Cancer Immunology and Virology, Dana-Farber Cancer Institute, Boston, MA 02215, USA; 3 Department of Biochemistry, University of Zurich, 8057 Zurich, Switzerland

**Keywords:** protein farnesyltransferase, site-specific protein conjugation, biorthogonal conjugation, targeted drug delivery

## Abstract

Protein-based conjugates have been extensively utilized in various biotechnological and therapeutic applications. In order to prepare homogeneous conjugates, site-specific modification methods and efficient purification strategies are both critical factors to be considered. The development of general and facile conjugation and purification strategies is therefore highly desirable. Here, we apply a capture and release strategy to create protein conjugates based on Designed Ankyrin Repeat Proteins (DARPins), which are engineered antigen-binding proteins with prominent affinity and selectivity. In this case, DARPins that target the epithelial cell adhesion molecule (EpCAM), a diagnostic cell surface marker for many types of cancer, were employed. The DARPins were first genetically modified with a C-terminal CVIA sequence to install an enzyme recognition site and then labeled with an aldehyde functional group employing protein farnesyltransferase. Using a capture and release strategy, conjugation of the labeled DARPins to a TAMRA fluorophore was achieved with either purified proteins or directly from crude *E. coli* lysate and used in subsequent flow cytometry and confocal imaging analysis. DARPin-MMAE conjugates were also prepared yielding a construct manifesting an IC_50_ of 1.3 nM for cell killing of EpCAM positive MCF-7 cells. The method described here is broadly applicable to enable the streamlined one-step preparation of protein-based conjugates.

## 1. Introduction

Since the approval of the first biologic, insulin, by the US Food and Drug Administration (FDA) in 1982, there have been enormous developments in the field of protein-based therapeutics. In addition to exploring various native proteins, significant efforts have been made to modify or conjugate proteins to achieve enhanced properties. For example, the attachment of a PEG polymer to a therapeutic protein has been widely employed to enhance the pharmacokinetics of the polypeptide [1,2,3]. As promising therapeutic agents in oncology, antibody-drug conjugates (ADCs) are generated by linking a small-molecule cytotoxin to the antibody so that toxic drugs can be selectively directed to malignant cells displaying targeted surface markers, thereby reducing systemic side effects [4,5,6,7,8]. Similarly, the incorporation of a metal chelator onto an antibody allows for the detection of tumorous tissue or even theranostic applications when a radioactive isotope is loaded [9,10,11]. In addition, the multivalent protein conjugates with enhanced avidity were also developed as potential drug carriers to deliver therapeutics for cancer treatment [12].

Due to the high demand for protein-based conjugates, a number of conjugation methods have been developed, including enzymatic labeling methods [13,14,15,16,17,18]. Taking advantage of the high efficiency and specificity of enzymes, homogeneously labeled product can be obtained. However, the introduction of an enzyme into the reaction mixture along with the target protein complicates the purification process. Moreover, the conjugation reaction may not achieve complete conversion, and in cases where the molecule being attached is small, as exemplified by most cytotoxic drug molecules, purification can be especially challenging since the physical properties of the modified protein differs only marginally compared to the unconjugated starting material. As a result, sophisticated chromatography methods are often needed which frequently require extensive optimization to achieve good separation. In one effort to simplify the purification process, solid microbeads were employed and functionalized with microbial transglutaminase (MTG) [19]. It was shown that the immobilized MTG was able to catalyze the conjugation of protein targets and could be easily filtered away from the product after reaction. However, this method still did not address the problem of purifying the conjugate from unmodified protein. To streamline the manufacturing process, a facile conjugation and purification strategy is highly desirable.

One such combined conjugation and purification strategy involves the use of the sortase A (SrtA) enzyme [20,21]. In this approach, a genetic fusion construct consisting of the target protein, followed by a sortag (for SrtA recognition) and an additional tag for purification, such as a His-tag, is employed. When the desired cargo is attached to the target protein by SrtA, the sequence downstream of the sortag is simultaneously cleaved off. Therefore, the conjugate can be easily separated from the unmodified protein based on the properties of the additional tag that is still attached to the unmodified protein. As a specific example, Policarpo et al. reported a flow-based SrtA ligation technique [22]. A micro-reactor was created by packing Ni-NTA resin pre-loaded with His-tagged SrtA enzyme. When the protein of interest, containing a C-terminal sortag and His-tag was added, it was first adsorbed onto the resin through the His-tag. With the addition of an oligo-glycine substrate for SrtA, the target protein was then modified with the substrate and eluted from the resin, while the unreacted protein remained attached to the resin through the His-tag. Although this design for the SrtA-based method is highly efficient, the conjugation process is still limited by the slow reaction kinetics and the moderate labeling yield of the wild-type enzyme [18]. Thus, high concentrations of the enzyme or the peptide substrates are usually required to achieve an acceptable yield. Although a penta-mutant variant with enhanced catalytic activity has been developed [23], it was shown to be associated with increased substrate hydrolysis in some cases [24]. In addition, when a solid support is utilized, optimization is still needed to minimize the formation of undesired side-products [25].

As an alternative enzymatic labeling technique, protein farnesyltransferase (PFTase) has also been utilized to create site-specific protein conjugates [26,27,28,29,30]. The enzyme catalyzes the transfer of an isoprenoid group from its native substrate, farnesyl diphosphate (FPP), to a C-terminal cysteine of the protein substrate. The recognition sequence for PFTase, consisting of only four amino acid residues, is denoted as the CaaX box, where C is the cysteine being modified. It has been shown that the incorporation of a C-terminal CVIA sequence onto a given protein of interest makes it a recognizable substrate for the enzyme, enabling efficient modification. In addition, a panel of isoprenoid analogues containing various bioorthogonal functional groups, including the azide [31], alkyne [32], aldehyde [33], and transcyclooctene [34] groups has been designed and synthesized, which can be used in lieu of FPP for conjugation [35]. In particular, our lab has previously reported a capture and release strategy based on the aldehyde functionality that can be used to streamline the protein conjugation and purification process [33]. In this technique, the protein of interest was first labeled with an aldehyde-containing substrate and immobilized onto hydrazide beads. Extensive washing of the beads allowed for the removal of the PFTase enzyme and any unmodified protein present. The release of the modified protein and conjugation to the desired cargos is accomplished by the addition of aminooxy-functionalized moieties, allowing one-step conjugation and purification. Proof-of-concept studies were conducted to modify GFP and a glucose-dependent insulinotropic polypeptide (GIP). A related aldehyde-based strategy was also employed using a carbonyl functional group that was incorporated into the target protein via chemical methods as recently described by Adusumalli et al. [15].

In this work, we applied the PFTase labeling method in concert with the aldehyde-based capture and release strategy to generate protein conjugates based on Designed Ankyrin Repeat Proteins (DARPins). Derived from natural ankyrin repeat proteins, DARPins are alternative binding scaffolds that can be engineered in vitro for selective target recognition with high affinity [36,37,38,39]. Compared to conventional antibodies, DARPins are small, highly stable and can be easily obtained from bacterial culture expression in high yield, as they have neither disulfides nor glycosylation sites. These favorable binding and physical properties make them extremely attractive for potential therapeutic or diagnostic applications [40,41,42,43,44,45,46,47].

In this study, we focused on several DARPin constructs designed to target epithelial cell adhesion molecules (EpCAM) [48], an antigen overexpressed on the cell surface of certain cancer cells [49]. Those proteins were appended with C-terminal CVIA sequences and subsequently enzymatically modified with an aldehyde-containing substrate using PFTase. Next, conjugation to a fluorophore was conducted using the capture and release strategy to yield functional DARPin-fluorophore conjugates, whose binding capability and selectivity were retained. DARPin-MMAE conjugates were also prepared and exhibited an excellent cytotoxicity profile in vitro. More importantly, labeling and conjugation could be achieved directly from crude cell lysates, which further simplified the production process, allowing for the one-step assembly and purification of functional protein-based conjugates without complicated chromatographic separation methods.

## 2. Results and Discussion

### 2.1. DARPin Modification by PFTase

Ac2 is a DARPin selected for its binding ability to EpCAM with low nanomolar affinity (biphasic dissociation was observed with *K*_d1_ of 2.2 nM and *K*_d2_ of 46 nM) [48]. The overexpression of EpCAM is observed on the cell surface in a variety of epithelium-derived tumors, including breast, pancreatic, and colorectal carcinoma, making it an attractive target for cancer diagnosis and therapeutics [50]. To enable site-specific modification, a C-terminal tetrapeptide sequence, CVIA, was genetically appended to Ac2. Three constructs were designed with different linker sequences between the DARPin and the CaaX-box to investigate whether additional spacer length is needed for enzyme recognition. Ac2-KCVIA (**D1**) has a single lysine residue as the spacer since that residue naturally precedes the CaaX motif in several Ras proteins, which are native substrates of PFTase [51]. Ac2-GSGTKCVIA (**D2**) contained a five-residue flexible linker. The third construct, Ac2-GGKKKKKKTKCVIA (**D3**), employed a poly-lysine segment derived from the C-terminal sequence of the K-Ras protein [51]. Enzymatic labeling reactions were performed with each of the protein constructs and the natural isoprenoid substrate, farnesyl diphosphate (FPP). The crude reaction mixtures were characterized by LC-MS equipped with a UV-Vis detector. Based on the LC-MS results, FPP modified Ac2 proteins were identified for each DARPin construct as the observed mass matched the calculated values (Figure S1). Therefore, all three proteins were successfully labeled by PFTase.

In addition to the product peak, a small peak that eluted before the modified DARPin was also detected in the A_280_ absorbance data from the LC chromatogram in each reaction. The mass of this small peak could not be deconvoluted due to low signal intensity (Figure S2). We attributed this peak to the unmodified DARPin, since the conjugation of a hydrophobic isoprenoid moiety typically causes a shift to a longer retention time of the labeled molecules compared to their unreacted counterparts on a reverse-phase column. To confirm the identity of this unknown peak, the starting material of each Ac2 construct was spiked into the reaction mixture and analyzed by LC-MS. As expected, the intensity of the unknown peak in each sample increased dramatically (Figure S2), suggesting that it was indeed the unreacted protein in the reaction mixture. Separation of the modified Ac2 from the unreacted starting material enabled quantification of the reaction yield by comparing the integrated peak areas from the A_280_ absorbance traces for both species. Using that approach, it was found that 87% of **D1** was labeled with FPP, while the yield was 86% for **D2** and 79% for **D3** (Figure S3). Therefore, all three constructs appear to be efficiently modified by PFTase and the linker length can be as short as one amino acid residue. To avoid potential non-specific interactions with the cell membrane from the highly positively charged poly-lysine residues in **D3**, **D1** was chosen for further investigation. As a negative control, E3_5 [52], a stable DARPin that does not bind to any target, was also engineered with a C-terminal KCVIA sequence (E3_5-KCVIA, **D4**). It was shown to be labeled by FPP with almost complete conversion, as observed from LC-MS analysis (Figure S4).

### 2.2. Enzymatic Incorporation of Aldehyde Functionality and Fluorophore Conjugation

**Scheme 1:**
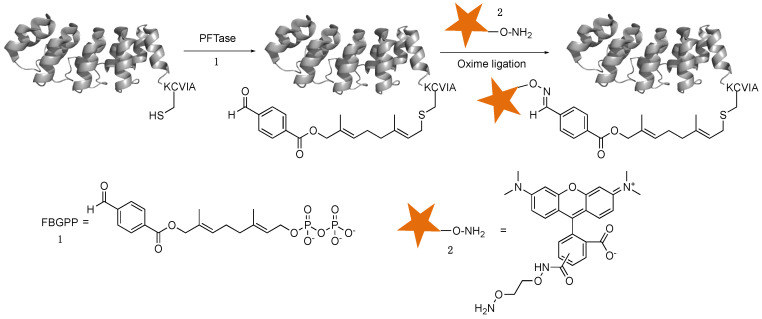
Site-specific enzymatic modification of DARPin with aldehyde **1** by PFTase and subsequent conjugation to TAMRA-aminooxy (**2**) using oxime ligation.

**Figure 1:**
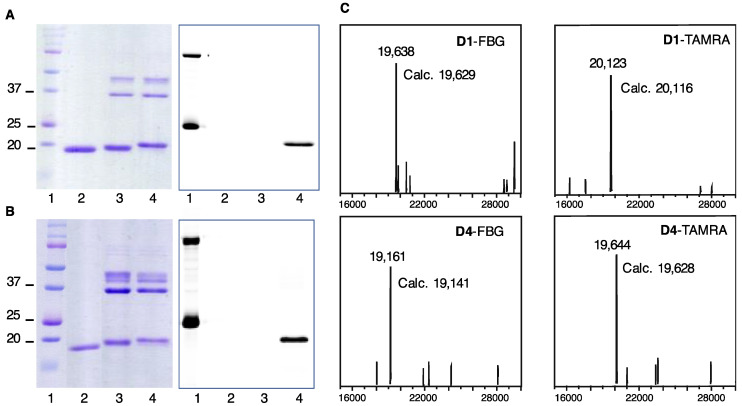
SDS-PAGE in-gel fluorescence imaging and LC-MS characterization of DARPin-FBG and DARPin-TAMRA conjugates. (**A**) SDS-PAGE (left) and in-gel fluorescence imaging (right) of **D1**-FBG and **D1**-TAMRA. Lane 1: protein ladder; lane 2: unmodified **D1**; lane 3: **D1**-FBG reaction mixture; lane 4: **D1**-TAMRA reaction mixture. (**B**) SDS-PAGE (left) and in-gel fluorescence imaging (right) of **D4**-FBG and **D4**-TAMRA. Lane 1: protein ladder; lane 2: unmodified **D4**; lane 3: **D4**-FBG reaction mixture; lane 4: **D4**-TAMRA reaction mixture. The bands around 37 kDa belonged to PFTase, which is a heterodimer. (**C**) MS of the modified DARPins. Upper left: Analysis of product from enzymatic modification of **D1** with FBGPP (**1**) to yield **D1**-FBG. Upper right: Analysis of reaction between **D1**-FBG and TAMRA-aminooxy (**2**) to yield **D1**-TAMRA. Lower left: Analysis of product from enzymatic modification of **D4** with FBGPP (**1**) to yield **D4**-FBG. Lower right: Analysis of reaction between **D4**-FBG and TAMRA-aminooxy (**2**) to yield **D4**-TAMRA. Note: For the gels shown in panels (**A**,**B**), some lanes have been removed in cases where they were duplicates or not relevant. However, all lanes shown in a given panel are from the same gel.

Once the enzymatic labeling of **D1** and **D4** by PFTase using FPP was validated, they were then modified with an aldehyde-containing FPP analog, formylbenzoyl-oxy geranyl diphosphate (FBGPP, **1**) (Scheme 1) [33]. As shown in Figure 1, successful aldehyde incorporation was achieved using both **D1** (Figure 1A, lane 3) and **D4** (Figure 1B, lane 3). The bands around 37 kDa in the SDS-PAGE can be attributed to PFTase, which is a heterodimer. Quantification of the A_280_ absorbance data from the LC-MS chromatogram suggested that the reaction yielded 88% conversion for **D1** and 95% for **D4** (data not shown). LC-MS analysis showed close agreement between the calculated and observed masses of the **D1**-FBG and **D4**-FBG products (Figure 1C, upper left and lower left). To create protein-fluorophore conjugates, the modified proteins were reacted with an aminooxy-containing fluorophore, TAMRA-aminooxy (**2**), using an oxime ligation reaction. Analysis via SDS-PAGE and in-gel fluorescence imaging showed strong fluorescent bands for the products (Figure 1A and B, lane 4), consistent with the formation of the desired conjugates. Interestingly, a small decrease in electrophoretic mobility was observed in the SDS-PAGE gel when **D1** was modified by **1** (Figure 1A, lane 3), while a further decrease was also noted after conjugation to **2** (Figure 1A, lane 4); similar results were observed for **D4** (Figure 1B). LC-MS was also used to confirm the mass of the DARPin-TAMRA conjugates (Figure 1C, upper right and lower right).

### 2.3. Capture and Release Strategy Allows Facile Construction of DARPin-TAMRA Conjugates

**Scheme 2:**
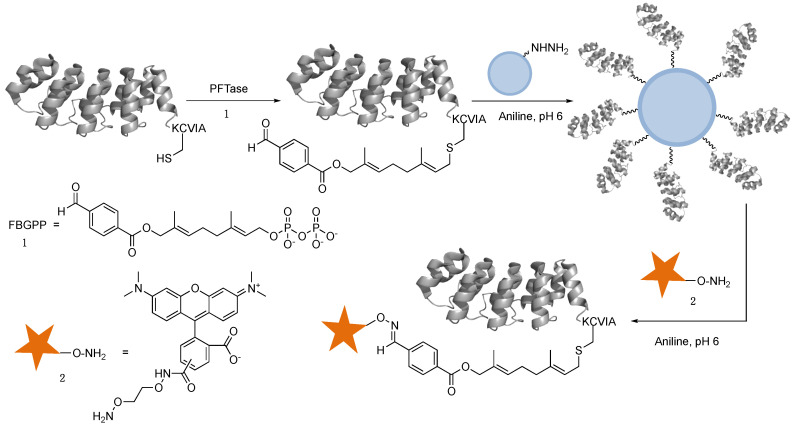
Capture and release strategy to construct DARPin-fluorophore conjugates.

**Figure 2:**
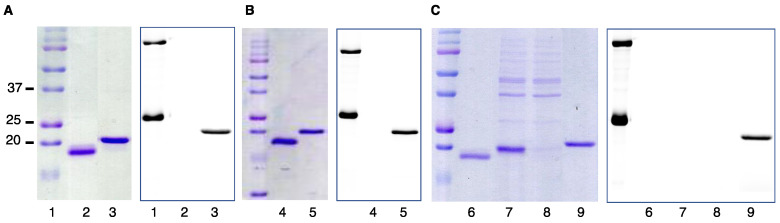
SDS-PAGE and in-gel fluorescence characterization of **D1**-TAMRA and **D4**-TAMRA constructed using capture and release strategy. (**A**) SDS-PAGE (left) and in-gel fluorescence (right) analysis of **D1**-TAMRA. Lane 1: protein ladder; lane 2: unmodified **D1**; lane 3: **D1**-TAMRA released from hydrazide beads. (**B**) SDS-PAGE (left) and in-gel fluorescence (right) analysis of **D4**-TAMRA. Lane 4: unmodified **D4**; lane 5: **D4**-TAMRA released from hydrazide beads. (**C**) SDS-PAGE (left) and in-gel fluorescence (right) analysis of **D1**-TAMRA from crude cell lysate. Lane 6: unmodified **D4**; lane 7: **D4**-FBG labeling mixture in crude lysate; lane 8: supernatant proteins that were not immobilized onto hydrazide beads; lane 9: **D4**-TAMRA released from the beads. Note: For the gels shown in panels (**A**–**C**), some lanes have been removed in cases where they were duplicates or not relevant. However, all lanes shown in a given panel are from the same gel.

To purify the DARPin-conjugates prepared from oxime ligation reactions, size-exclusion chromatography (SEC) can be employed to remove the larger PFTase enzyme (80 kDa). However, it is challenging to remove any unreacted DARPin from the corresponding conjugate, since the mass increase due to fluorophore installation is minimal compared to the molecular weight of the protein. To avoid multi-step chromatographic purification and complicated method optimization, a capture and release strategy was employed to generate and purify DARPin-TAMRA conjugates simultaneously (Scheme 2). In this method, the aldehyde modified DARPins were first captured onto hydrazide-functionalized beads through covalent hydrazone bond formation. The beads were then washed extensively to remove the PFTase enzyme and unmodified DARPins. Since the oxime bond is more stable than the hydrazone bond, the addition of aminooxy-containing compounds will drive the equilibrium towards the formation of the oxime product. Therefore, with the addition of excess **2**, the immobilized DARPins were eluted from hydrazide beads to form DARPin-TAMRA conjugates linked via oxime bonds. A simple desalting column was then utilized to remove excess small molecules including excess **2**, providing conjugates in pure form. Using this method, **D1**-TAMRA and **D4**-TAMRA were successfully prepared, as confirmed by in-gel fluorescence imaging analysis of the eluted materials (Figure 2).

To further streamline the process for protein conjugation, the capture and release strategy was applied to prepare DARPin-TAMRA conjugates directly from the crude cell lysate obtained from bacterial DARPin expression. Thus, after the induction of **D1** overexpression in *E. coli*, the cells were harvested and lysed. The concentration of **D1** in the lysate was estimated from a colorimetric protein assay in conjunction with densitometry from SDS-PAGE. The lysate was then supplemented with **1** and PFTase for enzymatic labeling. Since DARPin **D1** was the only protein present in the bacterial lysate containing a C-terminal CaaX-box sequence, it was the only protein prenylated with **1** by PFTase. Buffer exchange using centrifugal filters was performed to remove excess **1**, followed by the addition of the hydrazide beads. After immobilization and washing, compound **2** was added to elute the DARPin and form the **D1**-TAMRA conjugate. As shown in Figure 2C, **D1**-TAMRA could be successfully produced from cell lysate with high purity, leaving the impurities behind in the supernatant during immobilization. As a facile method to construct protein-based conjugates, the capture and release strategy eliminates the need for challenging chromatography purification steps to separate pure protein conjugates from unreacted starting materials. Furthermore, the ability to create protein conjugates directly from bacterial cell lysate further streamlines the production process, which can be easily adapted to large-scale manufacturing.

### 2.4. D1-TAMRA Retains Selective Binding to Cell-Surface EpCAM

**Figure 3:**
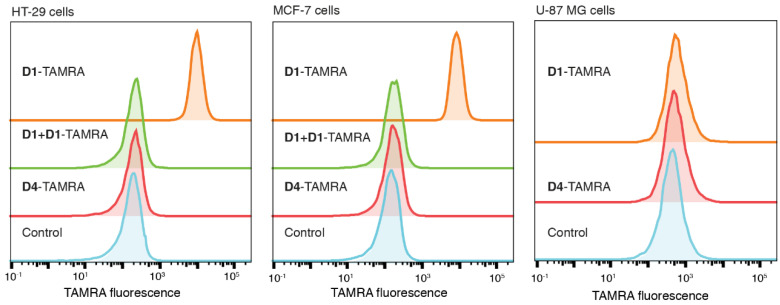
Evaluation of **D1**-TAMRA binding to cell-surface EpCAM by flow cytometry. HT29 and MCF-7 cells manifest EpCAM overexpression while U87-MG cells serve as a negative control. Left panel: Flow cytometry analysis of HT-29 cells treated with **D1**-TAMRA (orange, MFI = 9413), **D1**-TAMRA while pretreated with **D1** (green, MFI = 203), **D4**-TAMRA (red, MFI = 196), and vehicle control (blue, MFI = 173). Center panel: Flow cytometry analysis of MCF-7 cells treated with **D1**-TAMRA (orange, MFI = 8267), **D1**-TAMRA while pretreated with **D1** (green, MFI = 167), **D4**-TAMRA (red, MFI = 161), and vehicle control (blue, MFI = 136). Right panel: Flow cytometry analysis of U87-MG cells treated with **D1**-TAMRA (orange, MFI = 533), **D4**-TAMRA (red, MFI = 509), and vehicle control (blue, MFI = 436).

**Figure 4:**
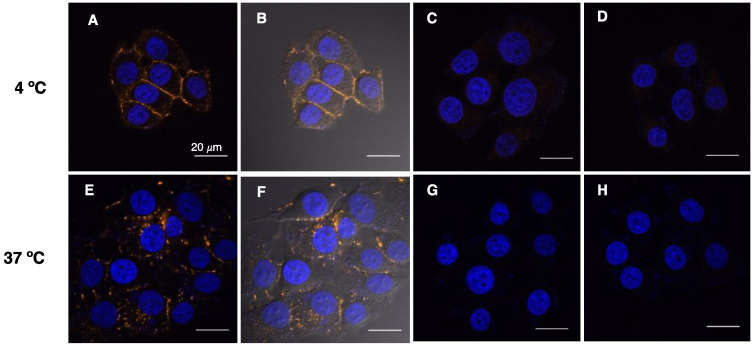
Visualization of binding (4 °C) and internalization (37 °C) of **D1**-TAMRA with MCF-7 cells studied by confocal microscopy. Cells were incubated with each construct for 1 h at 4 °C or 4 h at 37 °C, respectively. Panels (**A**,**E**): Cells treated with **D1**-TAMRA at 4 °C and 37 °C, respectively. Panels (**B**,**F**): Bright-field image of the cells treated with **D1**-TAMRA at 4 °C and 37 °C, respectively. Panels (**C**,**G**): Cells treated with **D4**-TAMRA at 4 °C and 37 °C, respectively. Panels (**D**,**H**): Cells without protein treatment at 4 °C and 37 °C, respectively. Blue: Hoechst stain for nucleus; orange: TAMRA fluorescence.

To investigate whether the modification interfered with the target binding capability of **D1**, flow cytometry experiments were performed using both EpCAM-positive (HT29 and MCF-7) and EpCAM-negative (U87-MG) cells. For HT-29 cells, treatment with **D1**-TAMRA gave a strong signal (Median Fluorescence Intensity, MFI = 9413, orange curve) that was 54-fold above the vehicle control sample (MFI = 173, blue curve). The **D1**-TAMRA signal was effectively reduced competed away by pretreatment with excess unlabeled **D1** (green curve), establishing that the cell surface association was **D1**-dependent. Similar results were observed for EpCAM-positive MCF-7 cells (Figure 3, center panel). In contrast, no sign of non-specific absorption was observed when **D1**-TAMRA was incubated with EpCAM-negative cells (Figure 3, right panel). As for the non-targeting **D4**-TAMRA, no cell-surface labeling was detected regardless of the EpCAM expression level, suggesting that the linker and the fluorophore did not cause nonspecific cell binding (Figure 3, left, center and right panels, red curves). For additional confirmation, the binding of **D1**-TAMRA to MCF-7 cells and subsequent internalization was visualized by confocal microscopy (Figure 4). When incubated with the cells at 4 °C, **D1**-TAMRA displayed clear cell-surface localization (Figure 4A). At 37 °C, the fluorescent signal from **D1**-TAMRA could be observed inside the cells in a punctate pattern, suggesting endosomal localization (Figure 4E). No cell surface localization or internalization was observed using **D4**-TAMRA (Figure 4C,G). Overall, these experiments provide compelling evidence that the selective target binding of **D1** was retained after conjugation to TAMRA.

### 2.5. Application of PFTase Labeling to a DARPin Binding another Target

**Figure 5:**
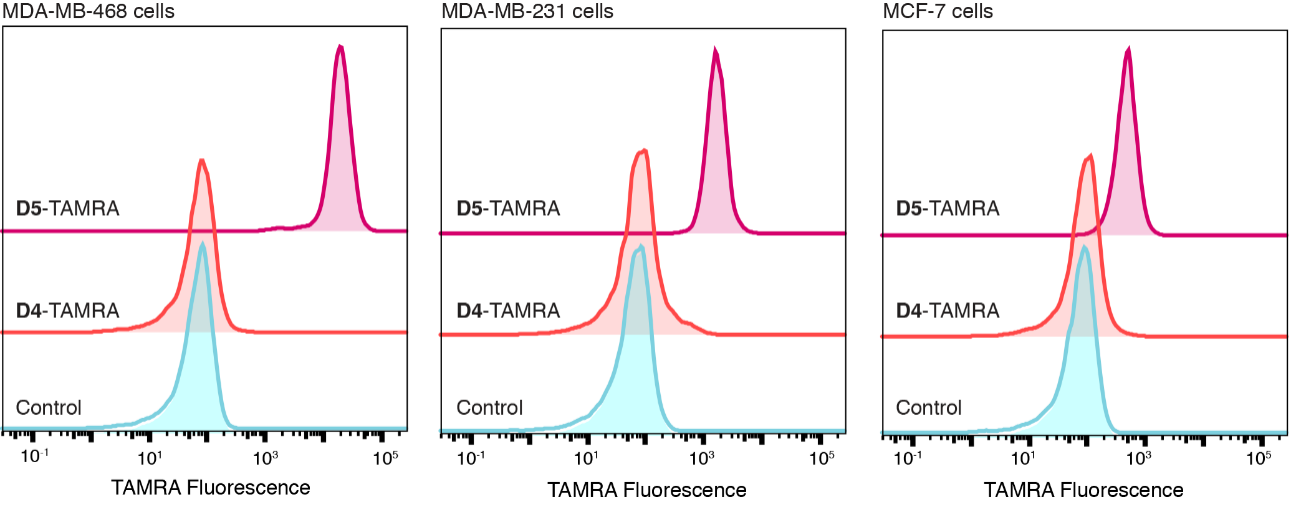
Evaluation of **D5**-TAMRA binding to cell-surface EGFR by flow cytometry using cells expressing EGFR at different levels. MDA-MB-468, MDA-MB-231 and MCF-7 cells express EGFR at high, medium and low levels, respectively. Left panel: Flow cytometry analysis of MDA-MB-468 cells treated with **D5**-TAMRA (pink, MFI = 18,968), **D4**-TAMRA (red, MFI = 74) and vehicle control (blue, MFI = 69). Center panel: MDA-MB-231cells treated with **D5**-TAMRA (pink, MFI = 1630), **D4**-TAMRA (red, MFI = 77) and vehicle control (blue, MFI = 64). Right panel: MCF-7 cells treated with **D5**-TAMRA (pink, MFI = 471), **D4**-TAMRA (red, MFI = 90) and vehicle control (blue, MFI = 76).

With the successful construction and purification of the EpCAM-binding DARPin **D1**, the applicability of this method to other DARPins was also explored. The DARPin E01 [53], which binds to the epidermal growth factor receptor (EGFR), a clinically validated cancer target, was selected and engineered to contain a C-terminal KCVIA sequence (E01-KCVIA, **D5**). Thus, **D5** was prenylated with the aldehyde-containing isoprenoid analogue **1**, followed by conjugation to a TAMRA fluorophore (Figure S5). Due to solubility issues with modified **D5**, the crude reaction mixture was utilized to evaluate whether the conjugated **D5** remained functional. **D5**-TAMRA was incubated with cells displaying various levels of total EGFR expression (MDA-MB-468, high; MDA-MB-231, medium; MCF-7, low) [54] and analyzed by flow cytometry (Figure 5). It was found that the fluorescence intensity detected from **D5**-TAMRA bound to cells correlated with their respective EGFR expression level, with the stronger signal corresponding to higher EGFR expression. MDA-MB-468 cells gave the strongest signal (MFI = 18,968) followed by MDA-MB-231 cells (MFI = 1630) and then MCF-7 cells (MFI = 471). The negative control, **D4**-TAMRA, did not show any non-specific binding towards any of the cell lines. These results confirmed that **D5**-TAMRA retained its binding selectivity towards cell-surface EGFR after labeling by PFTase and conjugation to TAMRA. Overall, the results support the idea that, as long as the binding/active site is not located in close proximity to the C-terminus, proteins engineered with the enzyme recognition sequence can be labeled by PFTase efficiently without affecting their functional activity.

### 2.6. Serum Stability of D1-TAMRA

**Figure 6:**
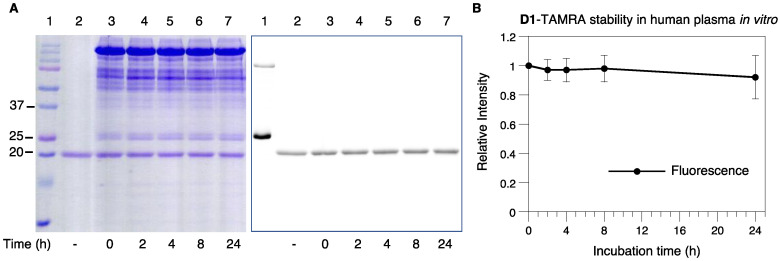
Serum stability of **D1**-TAMRA in vitro. Aliquots of **D1**-TAMRA diluted in 50% serum plasma were incubated at 37 °C for different durations and then flash-frozen. Each sample was tested in triplicate. Comparable results were achieved for the replicates with one of them shown as a representative. (**A**) SDS-PAGE (left) and in-gel fluorescence imaging (right) characterization of **D1**-TAMRA incubated in human plasma. Lanes 1: protein ladder; lanes 2: Purified **D1**-TAMRA without plasma incubation; lanes 3: 0 h incubation; lanes 4: 2 h; lanes 5: 4 h; lanes 6: 8 h; lanes 7: 24 h. (**B**) Quantification of **D1**-TAMRA fluorescence intensity in plasma samples at different time points. The band intensity was measured using Image J and plotted in Kaleidagraph. The error bars indicate the SD of three measurements.

To generate protein conjugates for potential clinical applications, the linkage between the protein and the cargo needs to be highly stable under physiological conditions. We therefore examined the stability of **D1**-TAMRA in serum plasma in vitro. First, **D1**-TAMRA was diluted in human plasma and incubated at 37 °C for different periods of time, including 2, 4, 8, and 24 h. SDS-PAGE and in-gel fluorescence imaging were employed to analyze the integrity of the conjugate, since the cleavage of the linker would result in the loss of the TAMRA fluorescence signal. As shown in Figure 6A, Coomassie blue staining following SDS-PAGE revealed that there were no additional bands in the DARPin molecular weight region (compare lanes 2 and 3), and the intensity of the **D1**-TAMRA band remained constant over time (see lanes 4–7) suggesting the protein was not proteolyzed during incubation. Additional confirmation came from quantification of the TAMRA fluorescence intensity that 90% of the TAMRA fluorophore was retained as the intact conjugate after 24 h of incubation (see Figure 6A, lane 7 and Figure 6B). Encouraged by these results, the incubation time was increased to 48 h, whereupon it was found that the conjugate maintained its stability (90%) (Figure S6). Thus, it appeared that the linker used to construct **D1**-TAMRA was stable in human serum plasma for at least 48 h.

### 2.7. Construction of DARPin-MMAE Conjugates

Since the labeled DARPins maintained their binding selectivity and affinity, we next constructed DARPin-based protein-toxin conjugates and evaluated their cytotoxicity in cell cultures. Monomethyl auristatin E (MMAE), a well-studied potent cytotoxic compound, was chosen as the warhead [55]. Since there are no commercially available aminooxy-modified MMAE derivatives, a DBCO-functionalized MMAE product (DBCO-MMAE, **4**) was utilized instead. Compound **4** contains the same cleavable linkage used in Adcetris, an FDA approved ADC developed by SeaGen, where the Val-Cit dipeptide is cleaved by lysosomal proteases to release the free MMAE drug [56].

To make it compatible with the DBCO functionality, two reaction schemes were designed to generate DARPin-MMAE conjugates using the capture and release strategy. In the first method (Scheme S1), an aminooxy-PEG-azide linker (**3**) was employed to react with **4** to form aminooxy-functionalized MMAE (**5**) using the strain-promoted azide-alkyne cycloaddition (SPAAC) reaction. The reaction mixture was directly added to the **D1** immobilized hydrazide beads to elute and form the **D1**-oxi-SPAAC-MMAE (**D1**-MMAE) conjugate. The eluted product was characterized by SDS-PAGE and LC-MS to confirm the successful formation of the **D1**-MMAE conjugate while removing PFTase from the final product (Figure S7). However, it should be noted that a large excess of compound **4** was consumed in this method to drive the elution process, which is not currently economically feasible for large-scale production with commercially available reagents.

**Scheme 3:**
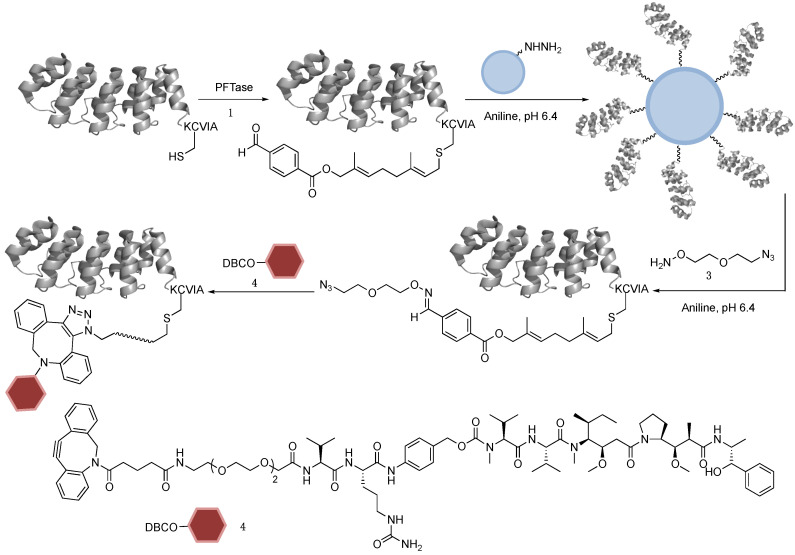
Construction of DARPin-MMAE conjugates using capture and release strategy. Compound **1** is FBGPP, the aldehyde-containing FPP analog. The immobilized aldehyde-modified DARPins were eluted using linker **3** to form DARPin-oxi-N_3_ conjugates, which were then reacted with **4** to yield the final DARPin-MMAE conjugates.

**Figure 7:**
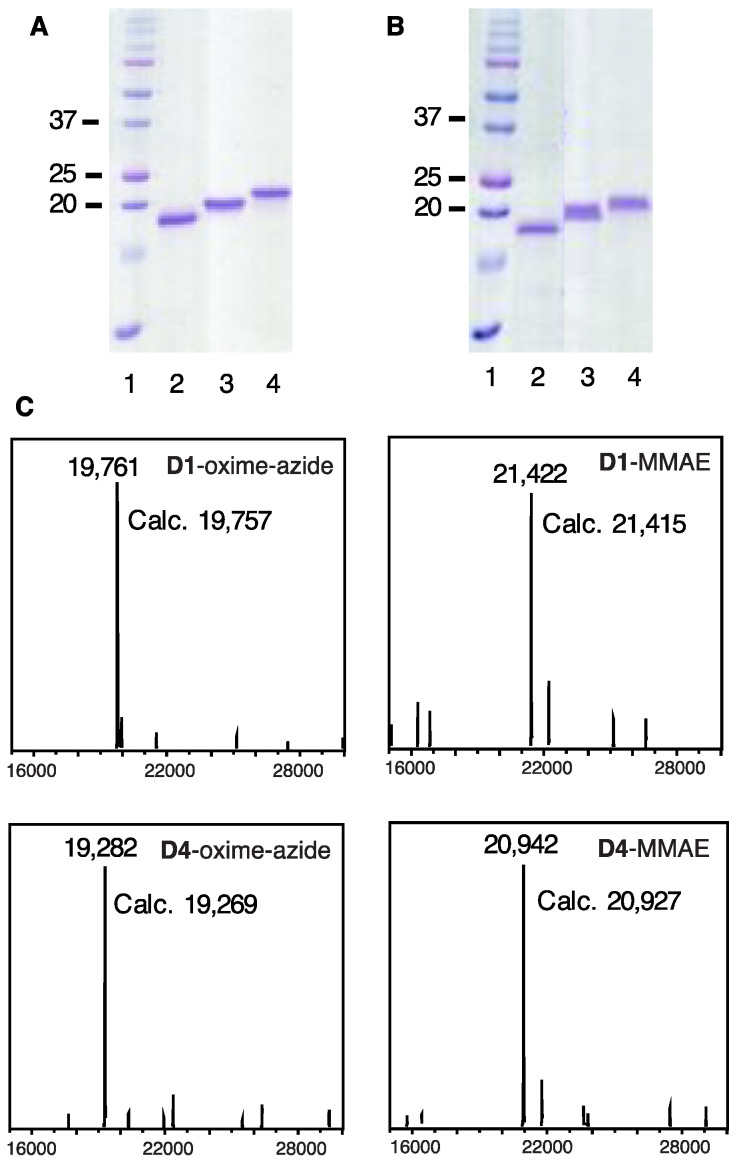
SDS-PAGE and LC-MS characterization of **D1**-MMAE and **D4**-MMAE. (**A**) SDS-PAGE of **D1**-MMAE. Lane 1: protein ladder; lane 2: unmodified **D1**; lane 3: **D1**-oxime-N_3_; lane 4: **D1**-MMAE. (**B**) SDS-PAGE of **D4**-MMAE. Lane 1: protein ladder; lane 2: unmodified **D4**; lane 3: **D4**-oxime-N_3_; lane 4: **D4**-MMAE. (**C**) Top panels: MS of **D1**-oxime-N_3_ and **D1**-MMAE; bottom panels: MS of **D4**-oxime-N_3_ and **D4**-MMAE. Note: For the gels shown in panels (**A**,**B**), some lanes have been removed in cases where they were duplicates or not relevant. However, all lanes shown in a given panel are from the same gel.

Therefore, a second strategy was utilized (Scheme 3). After the aldehyde-modified **D1** was immobilized onto hydrazide beads, it was eluted using linker **3** to form **D1**-oxime-N_3_. That azide containing molecule was then reacted with **4** in solution to yield the final **D1**-MMAE conjugates. It was found that simple desalting columns could not completely remove excess **4** from the conjugate, therefore, the SPAAC reaction mixture was purified by SEC chromatography. Using this method, several DARPin-MMAE conjugates were prepared, including **D1**-MMAE, **D4**-oxi-SPAAC-MMAE (**D4**-MMAE), and **D10**-oxi-SPAAC-MMAE (**D10**-MMAE). **D10** (Ec1-KCVIA) is an EpCAM-targeting DARPin modified with a C-terminal KCVIA sequence. **D10** binds a different epitope and features a higher binding affinity (K_d_ of 68 pM) compared with **D1** [48]; the selectivity of this higher affinity construct was confirmed by flow cytometry analysis using **D10**-TAMRA (Figures S9 and S10), and was subsequently used to investigate the impact of binding affinity on the cytotoxicity of the DARPin-MMAE conjugates. All the purified conjugates were characterized by SDS-PAGE and LC-MS. Based on the deconvoluted masses of the products, the successful formation of **D1**-MMAE, **D4**-MMAE (Figure 7), and **D10**-MMAE (Figure S11) was confirmed. High purity was confirmed through the assessment of the LC chromatogram monitored at 280 nm with minor amounts of unreacted azide-modified DARPin as the main impurities (Figure S8).

### 2.8. Cytotoxicity of DARPin-MMAE Conjugates In Vitro

**Figure 8:**
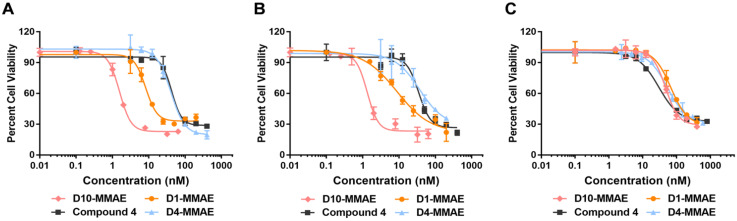
Cytotoxicity study of DARPin-MMAE conjugates using (**A**) HT29, (**B**) MCF-7, and (**C**) U87-MG cells. The cells were treated with different concentrations of DARPin-MMAE conjugates or compound **4** for four days. Cell viability was measured using an MTS assay. Panel (**A**): Cytotoxicity of DARPin conjugates in HT-29 cells. Panel (**B**): Cytotoxicity of DARPin conjugates in MCF-7 cells. Panel (**C**): Cytotoxicity of DARPin conjugates in U87-MG cells. Legend: **D10**-MMAE (pink, diamonds); **D1**-MMAE (orange, circles); **D4**-MMAE (blue, triangles); compound **4** (black, squares). Experiments were performed in triplicates.

The cytotoxicity of **D1**-MMAE and **D10**-MMAE was evaluated in cell culture using **D4**-MMAE as a control. Three cell lines were evaluated, including two that manifest EpCAM overexpression (HT29 and MCF-7) and one as negative control (U87-MG). Serial dilutions of DARPin-MMAE conjugates were prepared and added to the cells, whose viability was measured via MTS assay after 96 h of incubation. The results are plotted in Figure 8 and the IC_50_ values are summarized in Table 1.

As can be seen from Figure 8, both of the EpCAM-targeting DARPin-MMAE conjugates (**D1**-MMAE and **D10**-MMAE) were shown to be significantly more potent than the non-targeting **D4**-MMAE or the free drug molecule **4** in the EpCAM positive HT29 cells and MCF-7 cells. In HT-29 cells, **D1**-MMAE and **D10**-MMAE yielded IC_50_ values (see Table 1) of 8.3 and 1.6 nM, respectively, compared to 41 and 43 nM, for **D4**-MMAE and **4**, respectively. Thus, the EpCAM-binding DARPins showed substantially higher cytotoxicity compared with the untargeted DARPin or the toxin alone. **D1**-MMAE exhibited a five-fold increase in cytotoxicity compared with **4,** while **D10**-MMAE showed a 27-fold increase in cytotoxicity. Those results also highlight the impact of the higher affinity DARPin with **D10**-MMAE manifesting a five-fold improvement in cytotoxicity compared with **D1**-MMAE. Similar results were observed in MCF-7 cells. In contrast, in U87-MG cells, which do not express high levels of EpCAM, little difference was observed in the cytotoxicities of the various constructs, suggesting that the targeting of EpCAM is responsible for the greater potency of **D1**-MMAE and **D10**-MMAE.

The selectivity of these DARPin conjugates can also be characterized in a different way by comparing the toxicity of the same DARPin-MMAE conjugates in cells expressing high levels of EpCAM versus cells with minimal levels of the target to mimic the difference in tumor tissues versus normal tissues [57]. Thus, while **D1**-MMAE yielded an IC_50_ of 8.3 nM in HT-29 cells, it manifested a much higher value, 68 nM, when measured in U87-MG cells. As a result, **D1**-MMAE yields a selectivity ratio of 8. The same number was observed by comparing the cytotoxicity of **D1**-MMAE in MCF-7 cells with that measured in the control U87-MG cells. Importantly, using the higher affinity DARPin, **D10**-MMAE, the cytotoxicity selectivity improved; an IC_50_ of 1.3 nM was observed with **D10**-MMAE in MCF-7 cells versus 47 nM in U87-MG cells, yielding a selectivity value of 36. Similar results were obtained in HT-29 cells. The higher selectivity values exhibited by **D10**-MMAE are comparable to related antibody-drug conjugates assembled using similar linkers and the same drug payload [57], suggesting that **D10**-MMAE has excellent potential for use as a therapeutic agent for cancers characterized by EpCAM over-expression. Overall, the cytotoxicity data presented here demonstrate that DARPins can be site-specifically linked to cytotoxic drug molecules, resulting in conjugates with high cytotoxicity and selectivity against cancer cells in vitro.

## 3. Materials and Methods

### 3.1. Materials

The synthesis of aminooxy-functionalized TAMRA (**2**) is provided in the Supporting Information using a similar method described previously [58]. The TAMRA fluorophore was obtained from Biotium (Fremont, CA, USA). The isoprenoid analogue FBGPP (**1**) was synthesized as previously reported [33]. Compound **3** was purchased from BroadPharm (San Diego, CA, USA). Compound **4** was obtained from ACES Pharma, Inc (Princeton, NJ, USA). Other chemicals were obtained from MilliporeSigma (St. Louis, MO, USA). The protein assay dye reagent concentrate for the Bradford assay was purchased from Bio-Rad (Hercules, CA, USA). Dulbecco’s Modified Eagle’s Medium (DMEM), McCoy’s 5A (Modified) Medium, and fetal bovine serum were obtained from Thermo Fisher Scientific (Waltham, MA, USA). All the cell lines, including MCF-7, HT-29, U87-MG, MDA-MB-468, and MDA-MB-231 cells were generous gifts of Dr. Carston Wagner (Department of Medicinal Chemistry, University of Minnesota, Twin Cities). Yeast PFTase was purified as previously described [59]. The DARPins were purified using previously reported methods [48]. The LC-MS analysis was carried out using an Agilent 1100 series LC/MSD trap SL mass spectrometer (Agilent, Santa Clara, CA, USA) or an Orbitrap Elite Hybrid Mass Spectrometer (Thermo Fisher Scientific, Waltham, MA, USA) using Zebra 300 SB-C8 column (0.3 × 100 mm, 3.5 μm, Agilent, Santa Clara, CA, USA). The CellTiter 96^®^ AQueous One Solution Cell Proliferation (MTS) assay kits were from Promega (Madison, WI, USA).

### 3.2. Enzymatic Modification of DARPins

PFTase labeling reactions were performed in buffer containing: 50 mM Tris·HCl, 20 mM KCl, 10 mM MgCl_2_, 5 mM dithiothreitol (DTT), 10 μM ZnCl_2_, pH 7.5. The solution was incubated on ice for 0.5 h including 2.5 μM DARPins to reduce any disulfide bonds present. After incubation, 15 μM isoprenoid substrates were added to the solution, followed by the addition of 200–500 nM PFTase. The reaction was then transferred to a water bath at 32 °C and reacted for 6 h. LC-MS characterization was performed with the crude reaction mixture without further purification. To remove excess isoprenoid analogues, a buffer exchange using 1× PBS was conducted three times with Amicon filters (3K cut-off, MilliporeSigma, St. Louis, MO, USA).

### 3.3. DARPin-FBG Conjugation to TAMRA-Aminooxy (2)

Oxime ligation reactions were performed with 20 to 50 μM aldehyde modified DARPin-FBG and 5 eq of **2** at rt for 6 h on a rotary shaker. No catalyst was employed. A NAP-5 column (Cytiva, Marlborough, MA, USA) was used to remove excess **2** from the reaction mixture.

### 3.4. Capture and Release Strategy to Construct DARPin-TAMRA Conjugates

To immobilize the aldehyde-modified DARPins, 20 eq of UltraLink hydrazide beads (Thermo Fisher scientific Waltham, MA, USA, hydrazide loading: 15 μmol/mL), were pre-equilibrated with a 100 mM phosphate buffer, pH 6.4 and then added to the protein solution. The reaction was carried out in a 100 mM phosphate buffer, pH 6.4 with 5 mM DTT as well as 100 mM aniline as catalyst. After 2 h incubation on a rotary shaker at rt, the reaction mixture was centrifuged at 13,000× *g* for 2 min to remove the supernatant. The beads were washed with 10 bed volumes of 300 mM phosphate buffer three times, pH 6.4, followed by 10 bed volumes of 1 M NaCl for three times. The release of the DARPin-TAMRA conjugates was conducted in 100 mM phosphate buffer, pH 6.4, with 10 eq of **2** and 100 mM aniline as a catalyst. The elution reaction mixture was placed on a rotary shaker and allowed to proceed overnight. A NAP-5 column was employed to remove excess **2** from the conjugates using 1× PBS buffer.

### 3.5. Flow Cytometry Analysis of D1-TAMRA Binding to Cell Surface EpCAM

PBSA buffer containing 1x PBS with 1 mg/mL BSA protein was used here. Three different cell lines were analyzed including MCF-7 cells, HT-29 cells, and U87-MG cells. Cells were harvested and washed twice with PBSA. A total of 1 × 10^6^ counts/mL of cells (400 μL) were incubated with 100 nM **D1**-TAMRA and **D4**-TAMRA (diluted in PBSA), in the dark for 45 min at 4 °C. After incubation, cells were washed twice with PBSA before analysis. For competition experiments, cells were first incubated with 10 μM unlabeled **D1** at 4 °C for 15 min. Experiments were conducted using a BD LSR II/Fortessa H0081 flow cytometer (BD, Franklin Lakes, NJ, USA) and 1 × 10^4^ cells were counted. The data analysis was performed with FlowJo software (v10, BD, Ashland, OR, USA).

### 3.6. Visualization of D1-TAMRA Binding and Internalization to MCF-7 Cells

A total of 1 × 10^5^ MCF-7 cells were seeded onto sterile glass cover slips and incubated for 24 h. Fresh DMEM media containing 100 nM **D1**-TAMRA or **D4**-TAMRA was then supplied and incubated in the dark for 1 h at 4 °C or 4 h at 37 °C. After incubation, cells were washed for 3 min with 1× PBS three times and then fixed with 4% paraformaldehyde, followed by another wash with 1× PBS. The nuclei of the cells were stained with 0.5 μg/mL Hoechst 34,580 nuclear stain for 10 min at rt. After washing with 1× PBS, the cover slips were mounted on glass slides using SlowFade Diamond Antifade Mountant (Thermo Fisher Scientific, Waltham, MA, USA) and sealed with nail polish. Images were obtained using an Olympus FluoView FV1000 BX2 Upright Confocal microscope (Waltham, MA, USA). The analysis was performed with FluoView software (v4.2b, Olympus, Waltham, MA, USA).

### 3.7. Serum Stability of D1-TAMRA In Vitro

Human plasma (lithium heparin plasma, BioIVT, Westbury, NY, USA) was diluted in 1× PBS to 50% and filtered through a 0.2 μm filter. **D1**-TAMRA was then added to the 50% plasma to a final concentration of 1 μM. Aliquots of 160 μL were prepared and incubated in a 37 °C water bath for 0, 2, 4, 8, 24, and 48 h. At each desired time point, samples were removed from the water bath, flash frozen, and stored at −20 °C. Since **D1** has a His-tag at the N-terminus, 12 μL of Ni-NTA resin, pre-equilibrated with 1× PBS containing 20 mM imidazole, was added to the freshly thawed serum samples to capture the DARPins. The mixture was placed on a rotary shaker for 1 h in the dark at 4 °C. The supernatant was removed after centrifugation and the resin beads were washed twice with 1× PBS containing 20 mM imidazole. To release the bound protein, 20 μL of 1× Laemmli buffer with 1 M imidazole was added to the resin. The resulting mixture was incubated at rt for 10 min and then heated at 95 °C for 10 min. The supernatant was loaded onto an SDS-PAGE gel and subjected to electrophoresis. The experiment was performed in triplicate. In-gel fluorescence scanning was performed using a Typhoon FLA 9500 biomolecular imager (GE Healthcare, Chicago, IL, USA). The images were analyzed with ImageJ software (v1.51, NIH, Bethesda, MD, USA) and plotted in KaleidaGraph (v3.6, Synergy Software, Reading, PA, USA).

### 3.8. Construction of DARPin-MMAE Conjugates

The aldehyde-modified DARPins were immobilized onto hydrazide beads as described above. In route 1, compound **4** (50 eq of captured proteins) and **3** (10 eq of captured proteins) were reacted in DMSO at rt for 6 h. The reaction mixture was directly added to the DARPin-immobilized beads to form the desired DARPin-MMAE conjugates. The elution reaction was performed in a 100 mM phosphate buffer, pH 6.4 in the presence of 100 mM aniline. In route 2, the captured DARPins were eluted from the beads using 10 eq of **3** in 100 mM phosphate buffer, pH 6.4 in the presence of 100 mM aniline to generate the DARPin-oxime-N_3_ product. After overnight release, excess **3** was removed using a PD SpinTrap G-25 column (Cytiva, Marlborough, MA, USA). 1× PBS was utilized to elute the proteins from the column. Protein concentration was determined using a Bradford assay using BSA as standard. To perform SPAAC reactions, 10 eq of **4** was added to the DARPin-oxime-N_3_ solution. The reaction was carried out at rt overnight on a rotary shaker. A Superdex 75 increase 100/30 GL column (Cytiva, Marlborough, MA, USA) was used to remove excess **4** from the DARPin-MMAE conjugates since simple desalting columns did not provide sufficient resolution. Size exclusion chromatography was performed using a Knauer system (Berlin, Germany) employing 1× PBS as the running buffer with a flow rate of 0.4 mL/min. After purification, fractions containing the DARPin-MMAE were concentrated using Amicon filters (3K cut-off). The concentration of the conjugate was determined using a Bradford assay.

### 3.9. DARPin-MMAE Cytotoxicity Assay in Cell Cultures

To determine the cytotoxicity of DARPin-MMAE conjugates, an MTS assay was utilized. Three different cell lines were tested, including MCF-7, HT-29, and U87-MG cells. MCF-7 cells and U87-MG cells were cultured in Dulbecco’s Modified Eagle’s Medium (DMEM) with 4.5 g/L glucose and L-glutamine, supplemented with 10% fetal bovine serum (FBS), 100 U/mL penicillin, and 100 μg/mL streptomycin at 37 °C with 5.0% CO_2_. HT-29 cells were cultured in McCoy’s 5A (Modified) Medium with 3 g/L glucose and L-glutamine, supplemented with 10% FBS, 100 U/mL penicillin, and 100 μg/mL streptomycin at 37 °C with 5.0% CO_2_. Cells were seeded on sterile 96-well plates at a density of 2000 cells/well in 50 μL of the corresponding cell culture medium. After 24 h incubation, media containing serial dilutions of **D1**-MMAE, **D10**-MMAE or **D4**-MMAE was added so that the final volume in each well was 100 μL. A longer cell cycle was observed with MCF-7 cells; therefore, an incubation time of 96 h was chosen. After incubation, the media was removed from the plates followed by the addition of 20 μL of CellTiter 96^®^ AQueous One Solution Cell Proliferation Assay reagent (Promega, Madison, WI, USA) according to the manufacturer’s protocol on Promega (https://www.promega.com/-/media/files/resources/protocols/technical-bulletins/0/celltiter-96-aqueous-one-solution-cell-proliferation-assay-system-protocol.pdf (accessed on 20 September 2022)). Cells were incubated for 1 to 4 h before analysis. Absorbance at 490 nm was measured using a Bio-Tek Gen5™ Microplate Reader (Agilent, Santa Clara, CA, USA). Readings from untreated cells were used for normalization. The data was analyzed in Excel and plotted in Graphpad Prism (v8, Dotmatics, San Diego, CA, USA). Selectivity was calculated based on a reported method [57] by comparing the IC_50_ values of the same DARPin-MMAE conjugates in cells expressing high levels of EpCAM versus the control cell line.

## 4. Conclusions

In this work, several DARPins were engineered with a C-terminal KCVIA sequence and successfully labeled using PFTase. By adding the minimal four-residue recognition sequence required by PFTase along with a single lysine residue as linker, the potential perturbation to the structure, function and antigenicity of the DARPins was minimized. The use of readily synthesized isoprenoid analogues enables the incorporation of a variety of bio-orthogonal functional groups into DARPins, which can serve as reactive handles for subsequent conjugation. As a highly efficient enzymatic reaction, excellent labeling yields can be achieved using PFTase.

To simplify the production of protein-based conjugates, a capture and release strategy based on the aldehyde functionality and the differential equilibrium with hydrazide and aminooxy functionalities was employed. Using this approach, the unmodified protein and other impurities including the PFTase enzyme can be easily removed by simple washing steps, eliminating the need for complicated chromatographic procedures. More importantly, this strategy can be expanded to generate protein conjugates directly from cell lysates, which further streamlines the production process and can be easily scaled up. Two applications were demonstrated in this study using the capture and release strategy to construct DARPin-based conjugates. First, DARPin-fluorophore conjugates were created, which were shown to be capable of binding to their designated cell-surface targets selectively. Notably, the modification did not cause any non-specific association. The stability of the conjugates was evaluated in a human serum plasma sample, which was selected to mimic real physiological conditions. It was found that the DARPin-fluorophore conjugates were highly stable in human plasma for up to 48 h. As a second example, DARPin-MMAE conjugates were also prepared and evaluated in cell cultures. Limited by the availability and cost of the toxin reagent, an extra conjugation step was performed in solution to yield the DARPin-MMAE conjugates. The cytotoxicity results including one construct manifesting an IC_50_ of 1.3 nM for cell killing of EpCAM positive MCF-7 cells illustrate how this approach can be used to construct site-specific protein-drug conjugates with impressive potency and selectivity, demonstrating great potential for future therapeutic applications.

Overall, the combination of the enzymatic PFTase labeling method and the capture and release strategy can be broadly applied as a facile technique to generate various functionalized protein-based conjugates for a variety of biotechnological and pharmaceutical applications.

## Figures and Tables

**Table 1:** Cytotoxicity of DARPin-MMAE constructs and **4** against HT29, MCF-7, and U87-MG cells.

Molecule	IC_50_ (nM)			Selectivity Ratio *	
	HT29	MCF-7	U87-MG	HT-29	MCF-7
**4**	42.8 ± 6.2	34.8 ± 8.1	29.7 ± 3.9	n.a.	n.a.
**D4**-MMAE	40.6 ± 3.0	36.9 ± 5.7	53.6 ± 3.9	n.a.	n.a.
**D1**-MMAE	8.3 ± 0.8	8.8 ± 2.2	67.8 ± 4.1	8	8
**D10**-MMAE	1.6 ± 0.1	1.3 ± 0.2	47.4 ± 3.4	30	36

* Selectivity ratio was determined as the ratio between the IC_50_ values for U87-MG cells and either HT29 or MCF-7 cells.

## Data Availability

Data are contained within the article and Supplementary Material, and raw data are available on request from the corresponding author.

## Supplementary Materials

The following are available online at https://www.mdpi.com/article/10.3390/ijms231911537/s1.
