# Supplementary material for: Enzymatic Construction of DARPin-Based Targeted Delivery Systems Using Protein Farnesyltransferase and a Capture and Release Strategy
Source: Int J Mol Sci. 2022 Sep 29;23(19):11537. doi: 10.3390/ijms231911537 (PMC9569580; doi:10.3390/ijms231911537)
Supplement: Supplementary file 1 [file ijms-23-11537-s001.zip › ijms-1921075-supplementary.pdf]

Supporting Information

Enzymatic Construction of DARPin-Based Targeted Delivery Systems using Protein Farnesyltransferase and a Capture and Release Strategy

Yi Zhang,[1] Yiao Wang,[1] Safak Uslu,[2] Sneha Venkatachalapathy,[1] Mohammad Rashidian,[2] Jonas V. Schaefer,[3] Andreas Plückthun[3] and Mark D. Distefano[1]*

[1]Department of Chemistry, University of Minnesota, Minneapolis, MN, 55455, U.S.A.

[2]Department of Cancer Immunology and Virology, Dana-Farber Cancer Institute, Boston, MA, 02215, U.S.A.

[3]Department of Biochemistry, University of Zurich, Zurich, 8057, Switzerland

Table of Contents

Synthesis of TAMRA-aminooxy (**2**) *3*

Figure S1. MS of unmodified and FPP-labeled DARPin proteins *4*

Figure S2. TIC chromatograms of prenylation reactions with **D1**, **D2** and **D3** *5*

Figure S3. LC chromatograms monitoring A_{280} of the FPP prenylation mixtures of **D1**, **D2** and **D3** *6*

Figure S4. LC-MS characterization of **D4** prenylation reaction *7*

Figure S5. Characterization of **D5**-TAMRA conjugate *8*

Figure S6. **D1**-TAMRA stability in human serum *in vitro* for 48 h *9*

Scheme S1. Construction of DARPin-MMAE conjugates using a pre-formed aminooxy-functionalized MMAE reagent *10*

Figure S7. Characterization of **D1**-MMAE *11*

Figure S8. UV absorption Chromatograms at 280 nm of DARPin-MMAE conjugates from LC-MS *12*

Figure S9. Characterization of **D10**-TAMRA conjugate *13*

Figure S10. Flow cytometry analysis of **D10**-TAMRA *14*

Figure S11. MS characterization of the **D10**-MMAE conjugate *15*

Synthesis of TAMRA-aminooxy (2)

(5,6)-TAMRA, SE (0.019 mmol) was reacted with a di-aminooxy compound **S1**[1] (4 eq) in the presence of DIEA (8.5 eq) in DMF overnight at rt. The reaction mixture was purified by HPLC using a Zorbax 300 SB-C18 (5 µm, 9.4 x 250 mm, Agilent) column. Buffer A: H_2O with 0.1% TFA; buffer B: CH_3CN with 0.1% TFA. Gradient: 0 to 20% buffer B in 5 min, 20% buffer B to 35% buffer B in 45 min and then rise to 100% B in 5 min. Flow rate: 3 mL/min. Compound **2** eluted at approximately 22% buffer B.

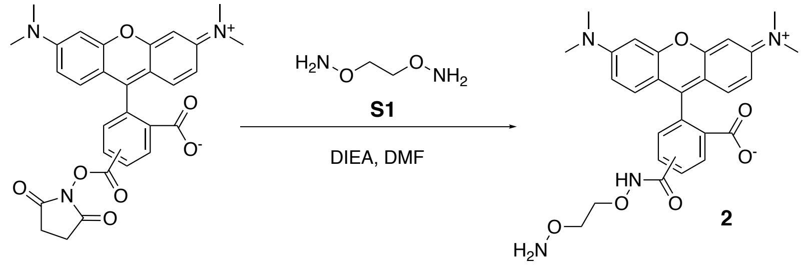

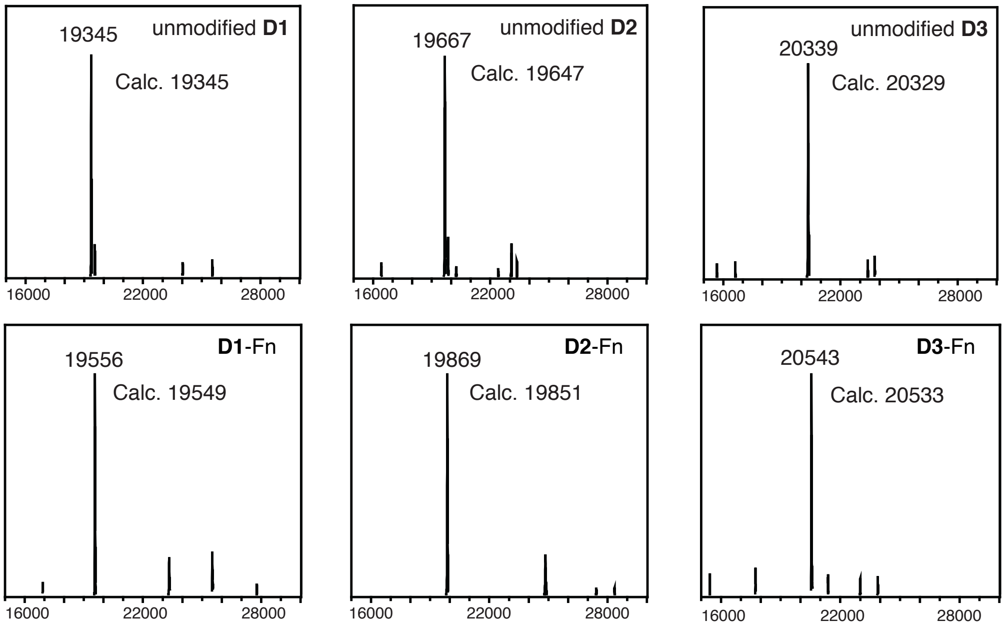

Figure S1. MS of unmodified and FPP-labeled DARPin proteins **D1**, **D2**, and **D3**.

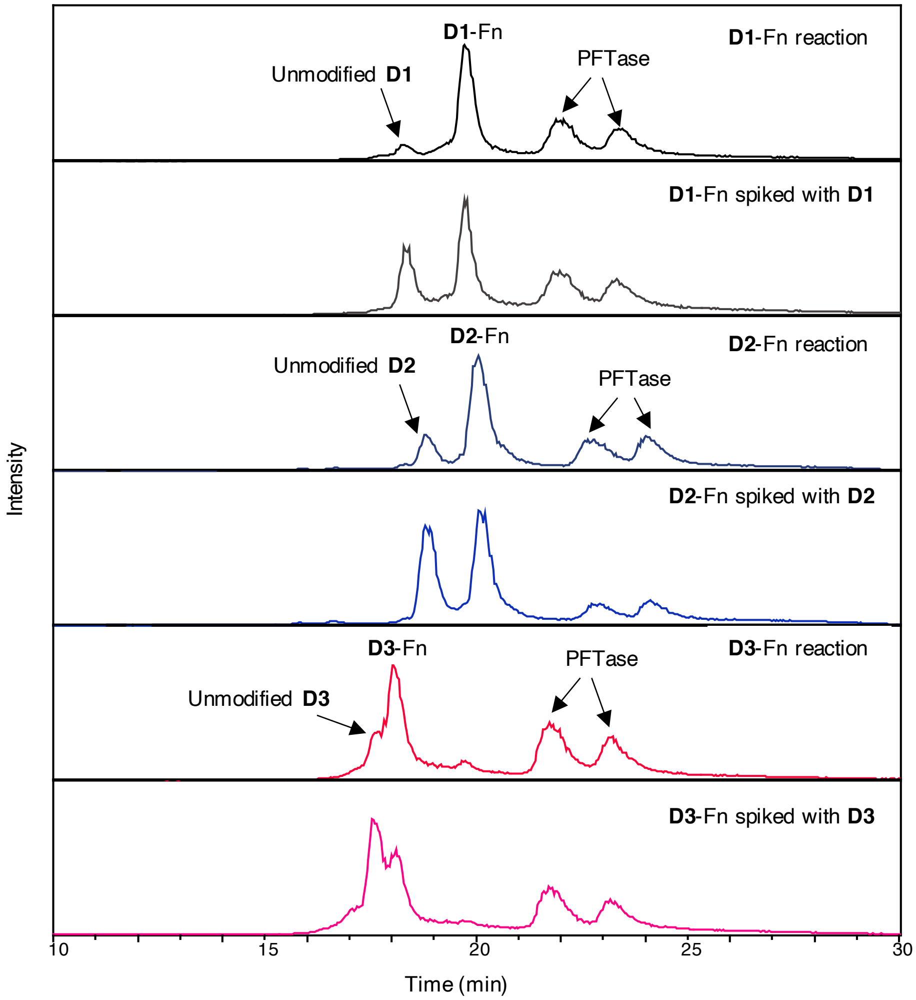

Figure S2. Total ion chromatograms (TIC) of prenylation reactions with **D1**, **D2**, and **D3** with FPP using PFTase. Unmodified protein was also spiked in the reaction mixture to help identify the peak for the unmodified protein in the reaction mixture.

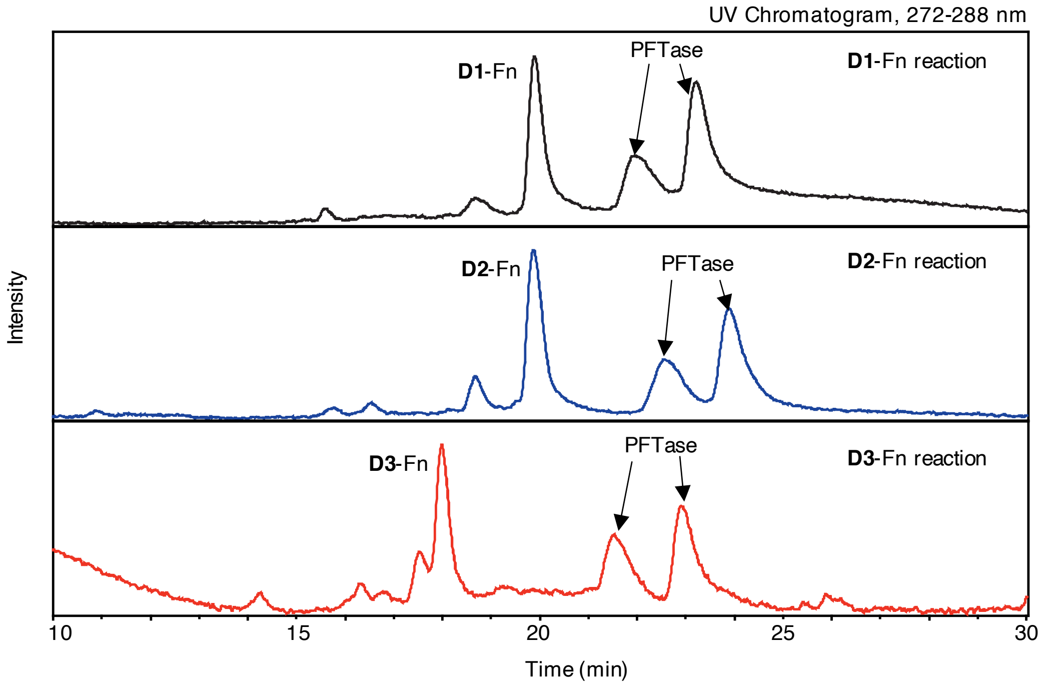

Figure S3. LC chromatograms monitoring A_{280} of the FPP prenylation mixtures of **D1**, **D2**, and **D3** for quantification of reaction yield from LC-MS characterization. Integration of the area in the A_{280} chromatogram was used to estimate the extent of conversion.

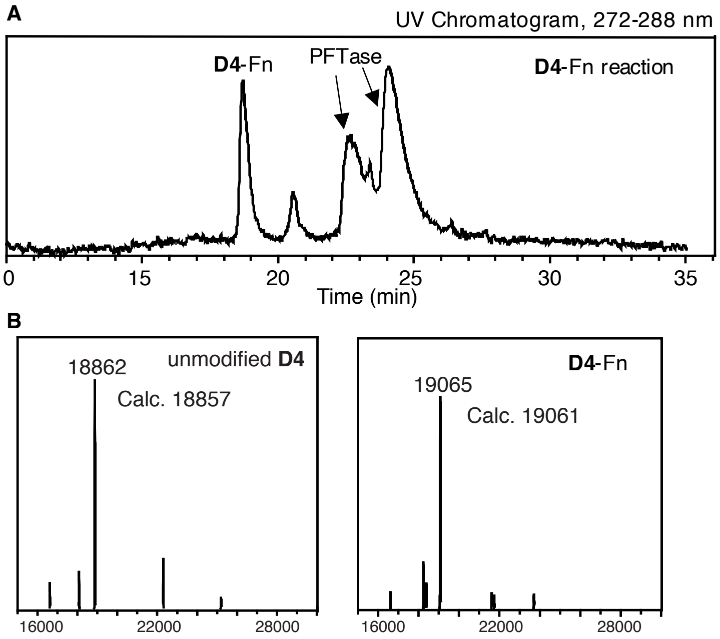

Figure S4. LC-MS characterization of **D4** prenylation reaction with FPP. (A) UV absorption Chromatogram at 280 nm of the reaction mixture. (B) MS of unmodified **D4** and **D4**-Fn.

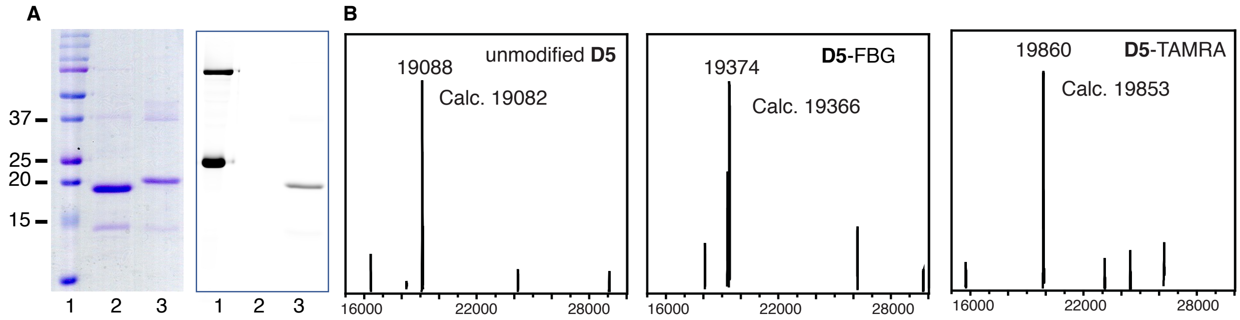

Figure S5. Characterization of **D5**-TAMRA conjugate. **(A)** SDS-PAGE (left) and in-gel fluorescence imaging (right) characterization of **D5**-TAMRA. Lane 1: protein ladder; lane 2: unmodified **D5**; lane 3: **D5**-TAMRA. The bands around 37 kDa belonged to PFTase, which is a heterodimer. (B) MS of unmodified **D5**, **D5**-FBG, and **D5**-TAMRA.

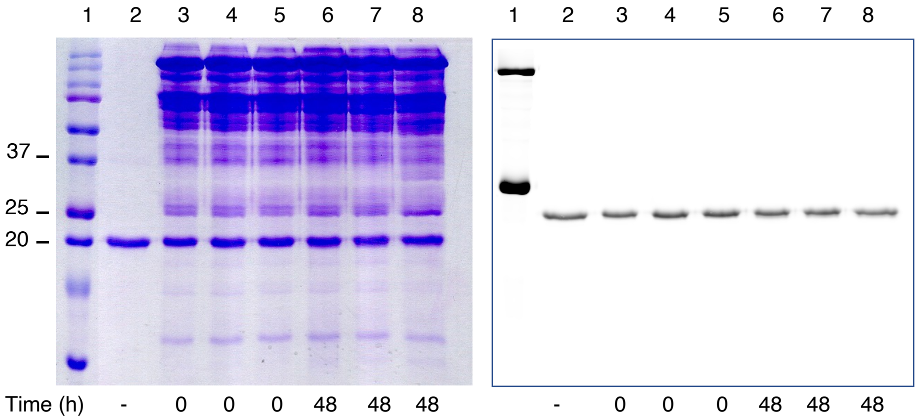

Figure S6. D1-TAMRA stability in human serum *in vitro* for 48 h. SDS-PAGE (left) and in-gel fluorescence imaging (right). Lane 1: protein ladder; lane 2: **D1**-TAMRA without plasma incubation; lanes 3 to 5: **D1**-TAMRA incubated in plasma for 0 h, three replicates; lanes 6 to 8: **D1**-TAMRA incubated in plasma for 48 h, three replicates. The two gel images were obtained from the same gel by first scanning for fluorescence (right image) followed by staining with Coomassie blue (left image).

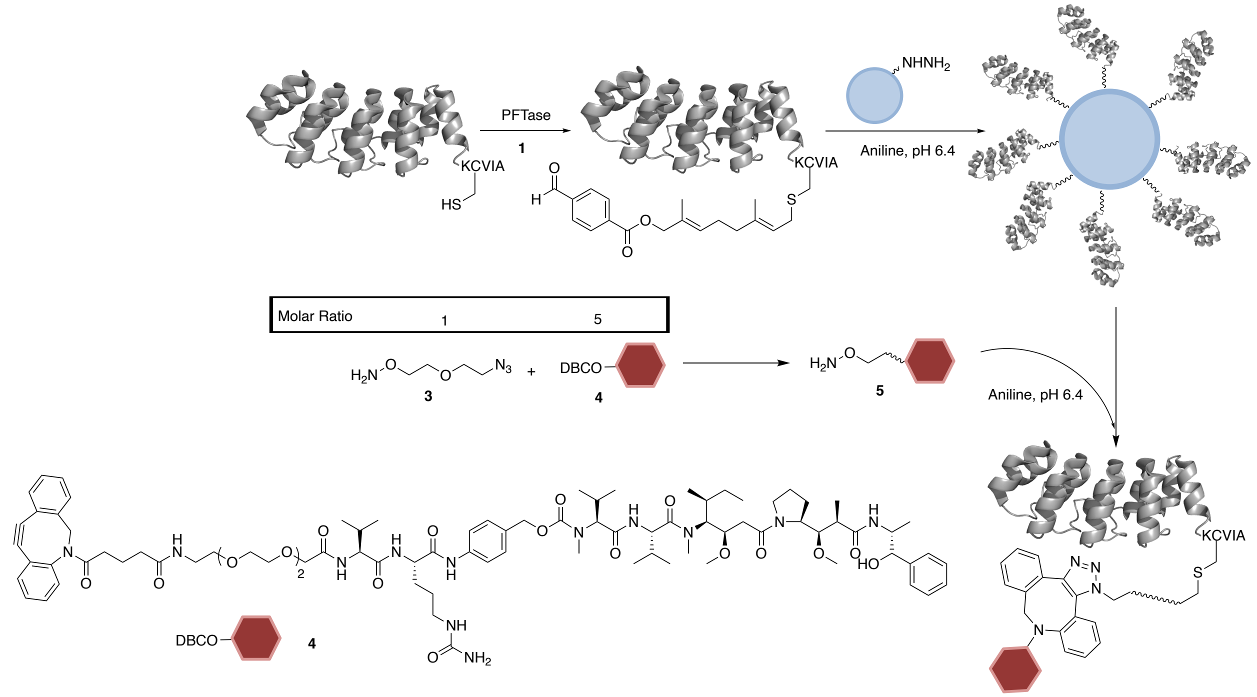

Scheme S1. Construction of DARPin-MMAE conjugates using a pre-formed aminooxy-functionalized MMAE reagent.

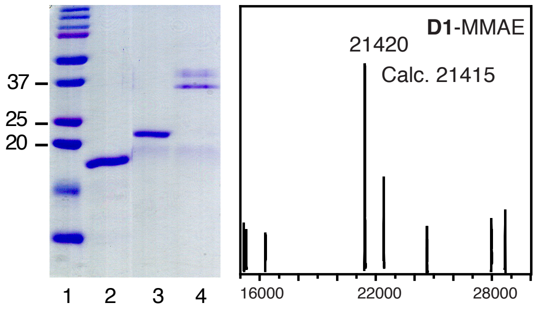

Figure S7. Characterization of **D1**-MMAE. SDS-PAGE (left) and MS (right) characterization of **D1**-MMAE prepared by the capture and release strategy using a pre-formed aminooxy-functionalized MMAE reagent. Lane 1: protein ladder; lane 2: unmodified **D1**; lane 3: **D1**-MMAE; lane 4: supernatant from the **D1**-FBG capture step showing that PFTase at higher molecular weight is removed in the capture process.

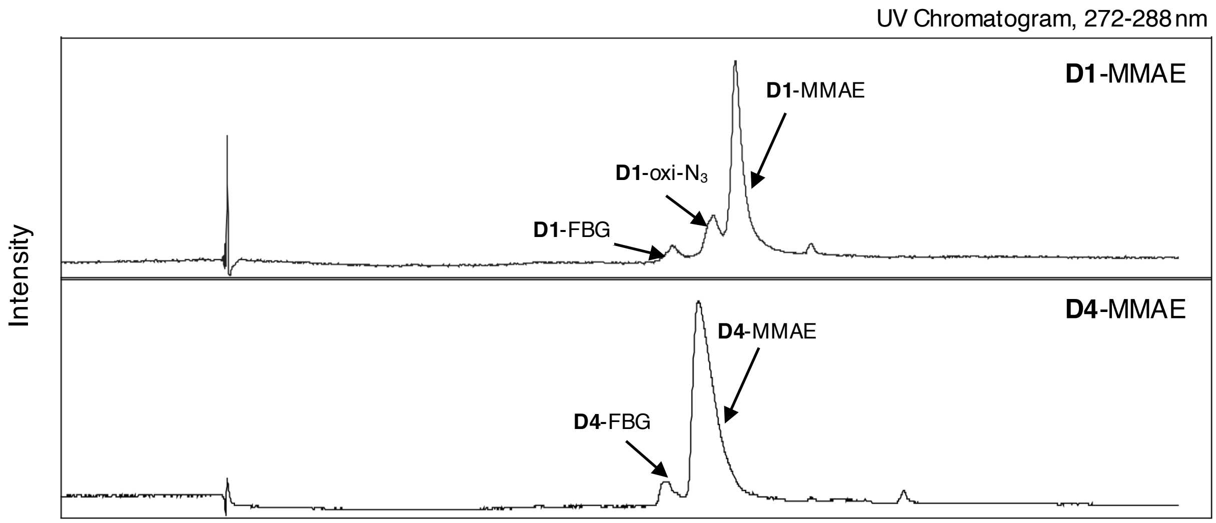

Figure S8. UV absorption Chromatograms at 280 nm of DARPin-MMAE conjugates from LC-MS to quantify the purity of **D1**-MMAE (77%) and **D4**-MMAE (93%) assembled using route 2.

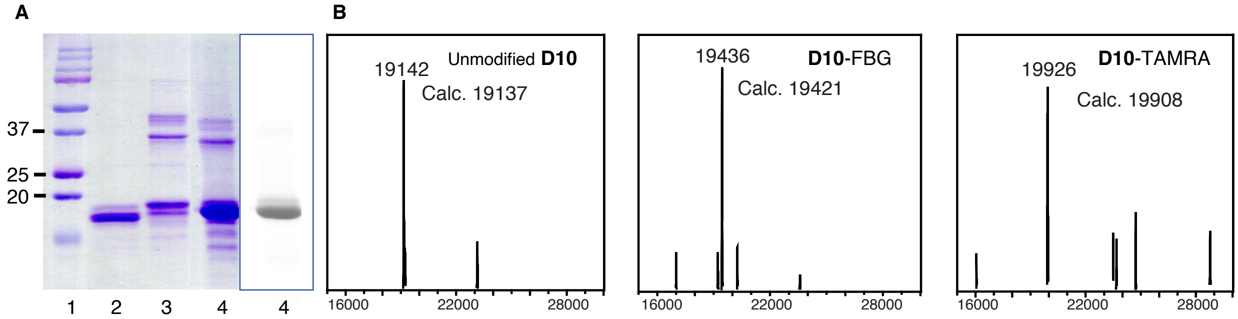

Figure S9. Characterization of **D10**-TAMRA conjugate. (A) SDS-PAGE (left) and in-gel fluorescence scanning (right) of **D10**-TAMRA conjugates. Lane 1: protein ladder; lane 2: unmodified **D10**; lane 3: **D10**-FBG reaction mixture; lane 4: **D10**-TAMRA reaction mixture. (B) MS of **D10** proteins including the unmodified protein, the protein modified with the aldehyde-containing substrate **1** (**D10**-FBG), and the protein after TAMRA conjugation (**D10**-TAMRA).

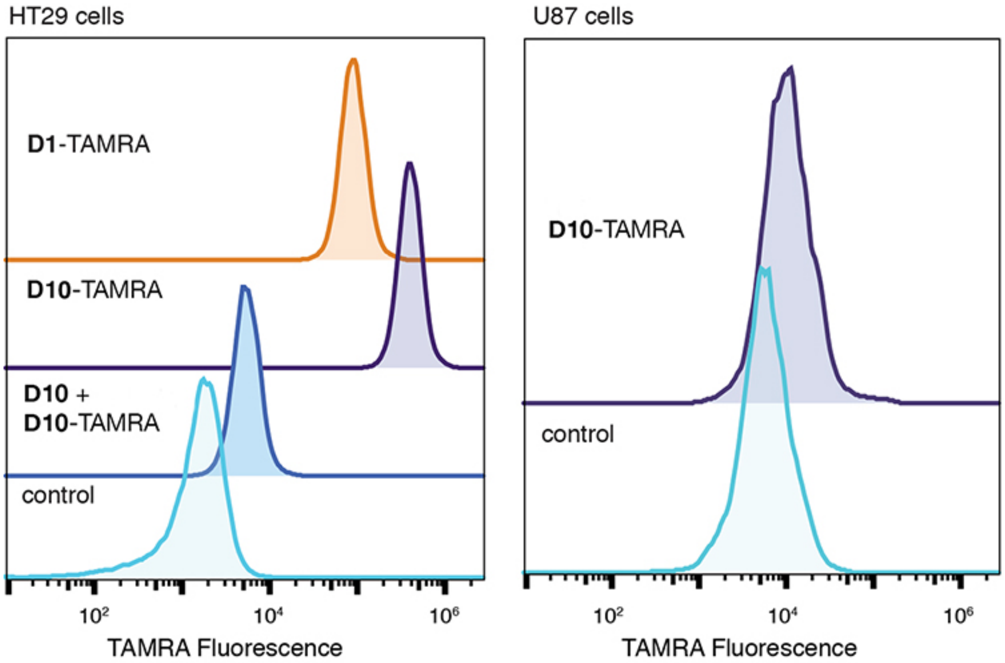

Figure S10. Flow cytometry analysis of **D10**-TAMRA binding to HT29 and U87-MG cells. Strong binding to EpCAM-positive HT29 cells were confirmed. Left panel: Flow cytometry analysis of HT-29 cells treated with **D1**-TAMRA (orange, MFI = 8,727), **D10**-TAMRA (purple, MFI = 38,705), **D10**-TAMRA pretreated with unmodified **D10** (blue, MFI = 536), and vehicle control (light blue, MFI = 164). Right panel: Flow cytometry analysis of U87-MG cells treated with **D10**-TAMRA (purple, MFI = 1,001), and vehicle control (light blue, MFI = 570).

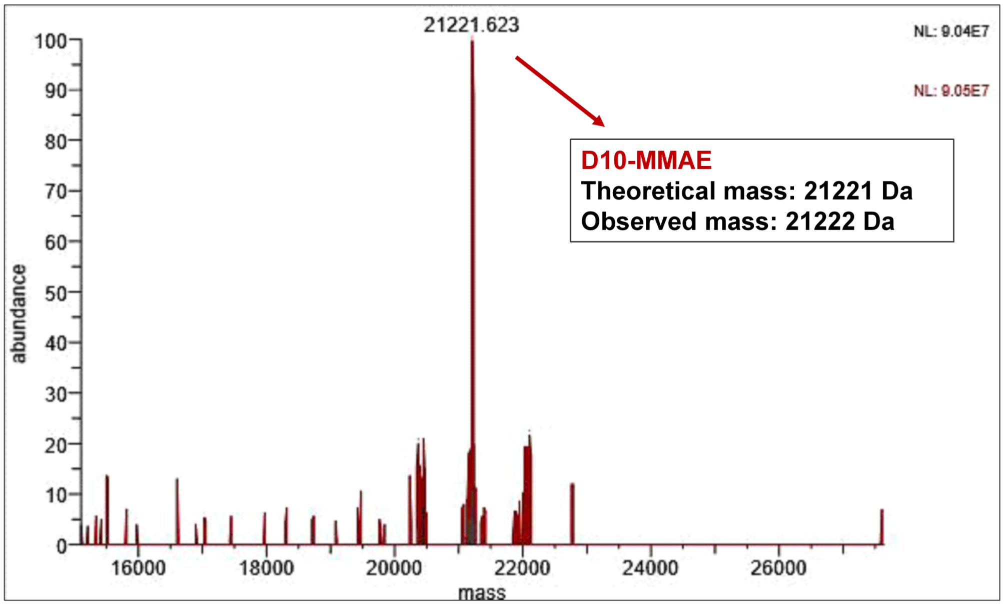

Figure S11. MS characterization of the **D10**-MMAE conjugate.

Reference

(1) Rashidian, M., Mahmoodi, M. M., Shah, R., Dozier, J. K., Wagner, C. R., and Distefano, M. D. (2013) A highly efficient catalyst for oxime ligation and hydrazone-oxime exchange suitable for bioconjugation. *Bioconjugate Chem. 24*, 333-42.
